# Positive and Negative Evidence Accumulation Clustering for Sensor Fusion: An Application to Heartbeat Clustering

**DOI:** 10.3390/s19214635

**Published:** 2019-10-24

**Authors:** David G. Márquez, Paulo Félix, Constantino A. García, Javier Tejedor, Ana L.N. Fred, Abraham Otero

**Affiliations:** 1 Department of Information Technology, Escuela Politécnica Superior, Universidad San Pablo-CEU, CEU Universities, Campus Montepríncipe, Boadilla del Monte, 28668 Madrid, Spain; 2 Centro de Investigación en Tecnoloxías da Información (CiTIUS), University of Santiago de Compostela, 15782 Santiago de Compostela, Spain; 3 Instituto de Telecomunicações, Instituto Superior Técnico, 1049-001 Lisboa, Portugal

**Keywords:** sensor fusion, clustering, evidence accumulation, fusion techniques, machine learning, ECG, multilead clustering, heartbeat clustering, multimodal clustering

## Abstract

In this work, a new clustering algorithm especially geared towards merging data arising from multiple sensors is presented. The algorithm, called PN-EAC, is based on the ensemble clustering paradigm and it introduces the novel concept of negative evidence. PN-EAC combines both positive evidence, to gather information about the elements that should be grouped together in the final partition, and negative evidence, which has information about the elements that should not be grouped together. The algorithm has been validated in the electrocardiographic domain for heartbeat clustering, extracting positive evidence from the heartbeat morphology and negative evidence from the distances between heartbeats. The best result obtained on the MIT-BIH Arrhythmia database yielded an error of 1.44%. In the St. Petersburg Institute of Cardiological Technics 12-Lead Arrhythmia Database database (INCARTDB), an error of 0.601% was obtained when using two electrocardiogram (ECG) leads. When increasing the number of leads to 4, 6, 8, 10 and 12, the algorithm obtains better results (statistically significant) than with the previous number of leads, reaching an error of 0.338%. To the best of our knowledge, this is the first clustering algorithm that is able to process simultaneously any number of ECG leads. Our results support the use of PN-EAC to combine different sources of information and the value of the negative evidence.

## 1. Introduction

The reduction in cost of consumer electronics and the ubiquity of wireless communication networks have enabled the use of devices to monitor physiological parameters, both for consumer and professional health applications [1,2]. Networks formed by several sensors have been extensively applied to body monitoring with a myriad of applications, ranging from fall detection to cardiovascular monitoring and gesture detection [3]. A fundamental issue when dealing with multiple sensors is how to combine them; some sensors can be imprecise due to noise or even incorrect due to the failure of the sensor. In some scenarios, sensors can generate redundant and/or highly correlated data, which can be a challenge itself, but it is also an opportunity to handle the noise of missing data. Furthermore, in the near future, the cheap availability of electronic components will allow for the strategy of using several sensors to measure the same physiological parameter, with the purpose of fusing the information coming from all of them to obtain higher quality information. Therefore, it is necessary to develop data fusion algorithms that combine the information of the different sensors to improve the monitorization of the patient [4].

Data fusion aims to integrate data from multiple sensors to create meaningful new information of higher quality than the one that could be extracted from an individual sensor, or even information that could not be obtained from any individual sensor. Dealing with multiple sensors usually requires specific scalable algorithms for sensor fusion [5]. Fusion and consensus problems have been applied in the following fields: fault diagnosis in mechanical systems [6,7], identification of human activities [8], human body tracking [9], and smart home systems [10], to name a few. However, this is still an open problem, given that more sensors, and consequently more signals, typically mean more dimensions in the feature space used to represent the problem. For example, in the case of the electrocardiogram (ECG), 12 different leads are typically recorded in the clinical routine. Each of these leads records the electrical activity of the heart from different points of view. Cardiologists interpret the data arising from each lead, compare, and combine them to reach the best possible diagnosis. However, the scientific papers dealing with ECG analysis typically use only one or two leads for the analysis of this signal. This is mainly due to the so-called “curse of dimensionality”: using more leads to represent each beat means using more features, and therefore more dimensions of the feature space, which typically hampers the performance of machine learning techniques.

Cardiovascular diseases are the number one cause of death in the world [11]. It is estimated that about 17.9 million people died from them in 2016, which represents 31% of all global deaths. Therefore, monitoring cardiovascular diseases has become a priority in many health systems. Electrocardiogram recording is the most broadly used method to do so, since it is inexpensive, non-invasive and effective. However, due to the large amount of data generated by the ECG, visual inspection is a tedious and time-consuming task. This is especially true in long recordings such as 24-h Holter, which can contain up to 100,000 heartbeats per lead, hence the interest in developing faster and better ECG analysis procedures. One of the first steps in the analysis of the ECG is the delineation and classification of the heartbeat according to its type: normal or ectopic beat (ventricular beat, escape beat, etc.). Grouping the heartbeats in the different types (clustering) has proved to be a valuable method to study the different arrhythmias and pathologies that affect the heart [12].

In the literature of heartbeat clustering, there is no clear consensus about the superiority of one clustering technique. It is possible to obtain similar results with a wide variety of techniques [12,13,14,15,16,17,18,19]. This is consistent with the comparative analysis done in the field of machine learning [20]; none of the clustering techniques has shown a clear advantage over the other techniques. Each clustering technique only presents a better adaptation to some specific types of problems.

**Figure 1:**
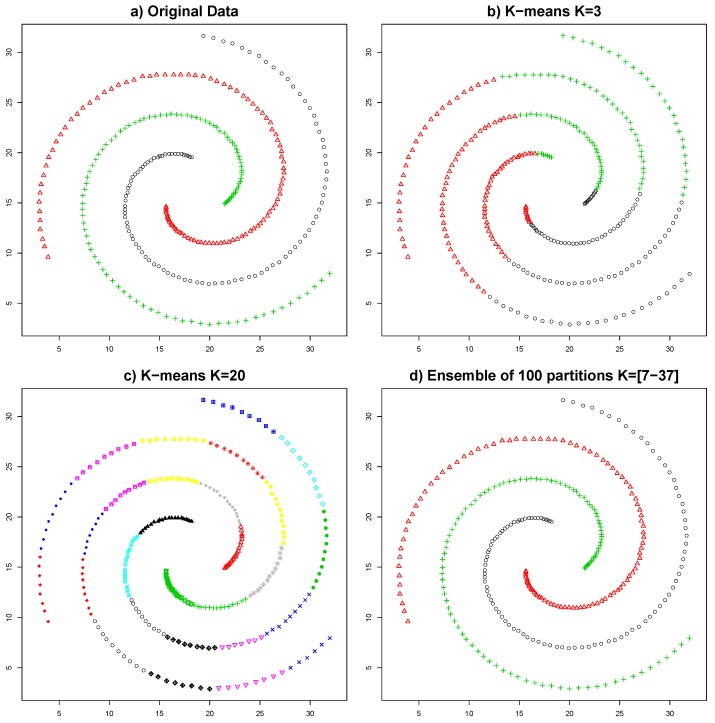
(**a**) shows the three original partitions of the data; (**b**,**c**) show two different executions of K-means with K = 3 and K = 20, respectively; (**d**) shows the result of the combination of 100 partitions generated by the K-means algorithm with random values for K between 7 and 37.

Recently, and taking inspiration from the works of classifier combination and sensor fusion, multiple techniques combining different clustering results have been proposed [21,22,23,24,25]. These clustering techniques, called *ensemble clustering*, have been established as a promising alternative to the traditional algorithms. The idea behind the ensembles is that the decision taken by a board of different experts is going to be more truthful than the decision taken by just one expert. One of their advantages is the capability of improving the robustness and the stability of the results by combining partitions generated from different algorithms, preserving their individual advantages and countering their individual limitations. For example, Figure 1 illustrates how combining the results of 100 executions of the K-means algorithm permits representing the three natural partitions of the data. Those partitions could never be obtained by a single K-means execution, given that this algorithm always obtains hyper-spherical partitions. An additional advantage of ensemble clustering is that it permits reducing the dependence between the clustering algorithm and the results, since different algorithms may be used and combined.

Evidence Accumulation Clustering (EAC) is one of the most used techniques for ensemble clustering. It was first proposed in [23] and is the result of a pursuit for new techniques that do not impose a fixed shape or model in the clusters. To this effect, it combines several clustering results, extracting information from each of them as partial evidence and combining this evidence to extract the final clusters. This idea has been extensively explored in classification, resulting in accuracy improvements by combining multiple classifiers [26,27,28]. In EAC, several individual clustering results are combined to obtain a new similarity measure that integrates and summarizes the information obtained from all the individual partitions. By using this new measure, it is possible to obtain the final partition of the data.

This paper proposes a novel ensemble clustering technique for sensor data fusion. This algorithm is based on the EAC paradigm and is able to combine an arbitrary number of sources of information. It also incorporates the concept of “negative evidence”, which permits the exploitation of information that is typically discarded in traditional evidence accumulation algorithms, which only rely on positive evidence. Positive evidence provides information on those elements that should be grouped together, whereas negative evidence provides information on those elements that should not be grouped together. The main contributions of this paper are:

- The introduction of a new concept called negative evidence, which permits exploiting information about those elements that should not be grouped together, information which is usually discarded by traditional ensemble clustering approaches.
- The validation of this new concept over a database comprising 109,966 heartbeats manually labelled by cardiologists (the MIT-BIH Arrhythmia Database), showing how a lower error (statistically significant) can be obtained when the concept of negative evidence is included, compared with traditional ensemble clustering approaches based only on positive evidence.
- A validation over an increasing number of data sources (from 2 to 12 ECG leads of the St. Petersburg Institute of Cardiological Technics 12-Lead Arrhythmia Database database (INCARTDB), which comprises 165,514 manually labelled heartbeats), where it is shown how, when the number of data sources increases, our algorithm obtains better results (statistically significant) than with a lower number of leads. The algorithm used in this validation is, to the best of our knowledge, the first algorithm capable of performing clustering over any number of ECG leads.

The rest of the paper is organized as follows: Section 2 presents the novel algorithm and shows how it can be applied to robust heartbeat clustering by combining the information provided by the different ECG leads. This information does not only comprise morphological features of the heartbeats, but also rhythm features. The experimental setup for the validation over the MIT-BIH Arrhythmia Database and the INCARTDB database is described in Section 3. Results of the validation are presented in Section 4, which are further discussed in Section 5. Finally, conclusions are provided in Section 6.

## 2. Positive and Negative Evidence Accumulation Clustering (PN-EAC)

For convenience, we refer to the proposed algorithm as Positive and Negative Evidence Accumulation Clustering (PN-EAC). The first step in the algorithm is to obtain the different data partitions. Each of these partitions can be obtained from a different data source (for example, measurements from a different sensor). Afterwards, evidence is extracted from each individual partition. In particular, we extract the information about the co-occurrence of two elements in the same cluster among the different partitions. This is normally referred to as evidence in the bibliography, but we shall call it *positive evidence*. Positive evidence provides information about the elements that should be grouped together in the final partition. A novel aspect of PN-EAC is that it is not limited to using positive evidence, but it can also handle information regarding the elements that should not be grouped together in the final partition; we shall call this information *negative evidence* [29].

The different steps of the PN-EAC algorithm (see Algorithm 1) are explained in detail in the following sections. First, how the partitions $P_{1},P_{2},\ldots ,P_{m}$ are generated is explained, then how these partitions are combined, and finally how the final partition $P_{*}$ is extracted. Let $X=\{x_{1},x_{2},\ldots ,x_{i},\ldots ,x_{n}\}$ be a finite set of *n* elements where each $x_{i}$ is a feature vector that represents the element *i*. A clustering algorithm will take *X* as input and will generate a data partition $P=\{A_{1},A_{2},\ldots ,A_{k}\}$ of the *n* elements of *X* divided in *k* groups, where $A_{j}$ represents the *j*-th group of the data partition. An ensemble is defined as a collection of *m* different data partitions $E=\{P_{1},P_{2},\ldots ,P_{m}\}$, obtained using *m* different clustering algorithms, different parameters or different data representations. No assumption is made about the number of groups in each partition of the ensemble; i.e., each partition could have a different number of *k* groups. The partitions of the ensemble are combined in a final partition, $P_{*}$, which is the final result of the evidence accumulation clustering and that, in general, will be more similar to the natural partition of the underlying data structure than most of the individual partitions separately. The technique also permits more expressiveness in the final results than each of the individual clustering algorithms, overcoming their limitations in the geometry and shape of the clusters.

**Algorithm 1** General scheme of the PN-EAC algorithm.**Require:** Set of elements $X=\{x_{1},x_{2},\ldots ,x_{n}\}$**Ensure:**$P_{*}$ final partition of the *n* elements
1:Generate data partitions $P_{i}$ with the *n* elements of *X*
#Section 2.1
2:Extract and combine positive and negative evidence from the partitions $P_{i}$
#Section 2.2
3:Generate the final data partition $P_{*}$
#Section 2.3

### 2.1. Generation of the Partitions of the Ensemble

When using clustering techniques based on ensembles, it is necessary to generate different data partitions $P_{1},P_{2},\ldots ,P_{m}$ (*m* is the number of data partitions from the data set). The use of different representations of the data (possibly measured by different sensors), techniques to alter the data (like sampling or bagging [30]), different clustering algorithms or different parametrizations of those algorithms, will produce, in general, different partitions of the same data. Ensembles can overcome the limitations of the individual results, abstracting the different representations, models or initializations and extracting the mutual information of the different data partitions.

In the myriad of clustering methods, the K-means algorithm stands out as the most commonly used. The simplicity of this algorithm is one of its major advantages, being easy to understand, computationally efficient, and fast. Its reduced number of parameters (typically only the number of clusters) is another of its strengths. Its major limitation is the inflexibility on the clusters that it forms, which always have a hyper-spherical shape. This limitation makes impossible the identification of clusters with arbitrary shapes, making it unsuitable for many applications which need to model complex shapes. However, as shown in [31], by combining several executions of the K-means algorithm, it is possible to overcome this limitation (see Figure 1) and represent clusters of virtually any shape.

In this work, K-means will be used to generate the data partitions. This algorithm is used due to its efficiency and its reduced computational cost. K-means is executed several times with arbitrary initializations for the initial centroids. The number of clusters *k* for each execution is obtained randomly in a given range; this value should be small compared with the number of elements *n* in the data set, but large enough to capture the different underlying groups. In this work, we shall adopt a common rule of thumb used by other ensemble clustering algorithms where this range is given by [23]:

(1) $$k\in \left[\sqrt{n}/2,\sqrt{n}\right],$$

where *n* is the number of elements. Given that K-means is a greedy algorithm, it is possible to obtain different results if the initialization is different, even for the same *k*.

### 2.2. Extraction and Combination of Positive and Negative Evidence from the Partitions

In the ensemble clustering literature, it is possible to find several approximations to combine the results of the different data partitions [25,32,33]. This work adopts the proposal of [23] based on evidence accumulation. This approach uses a voting mechanism to obtain the information of those pairs of elements that appear in the same cluster. The voting mechanism makes no assumptions about the individual partitions, making it possible to combine partitions with different numbers of clusters or even incomplete partitions (i.e., partitions without all the elements).

Positive Evidence Extraction

A subset of the original *m* partitions comprises the positive evidence partitions. Let $E^{+}=\left\{P_{1}^{+},P_{2}^{+},\ldots ,P_{q}^{+}\right\}$, $E^{+}\subset E$, be the ensemble of data partitions used to extract positive evidence, where *q* is the number of positive evidence partitions.

The information extracted by the voting mechanism will be gathered in a matrix $G^{+}$, whose size is n×n (*n* is the number of elements). For each individual partition, the co-occurrence of a pair of elements *i* and *j* in the same cluster will be treated as a positive vote in the corresponding cell of the $G^{+}$ matrix. The underlying idea is that those elements that belong to the same “natural” cluster will most likely be assigned to the same cluster in the different data partitions $P_{1}^{+},P_{2}^{+},\ldots ,P_{q}^{+}$. The $G^{+}$ matrix, considering the *q* data partitions, will be:

(2) $$G_{\left(i,j\right)}^{+}=\frac{q_{ij}}{q},$$

where $q_{ij}$ is the number of times that the pair of elements *i* and *j* has been assigned to the same cluster in the different data partitions $P_{1}^{+},P_{2}^{+},\ldots ,P_{q}^{+}$.

Negative Evidence Extraction

In some cases, due to the data representation used, the fact that two elements belong to the same cluster of a data partition can provide little information about the natural grouping of the data. This evidence could even introduce noise in the evidence matrix, worsening the final result. For example, let us suppose we want to group different types of physical activities in a set of subjects, and that we have inertial sensors and electromyographic sensors located in various parts of the subjects’ body, including their forearms. In this context, recording similar data from the forearm inertial sensors of two subjects provides evidence that subjects are performing similar actions with their forearms (positive evidence). However, presenting a similar electromyographic activity provides very weak evidence that the subjects are performing the same action. Riding a bicycle (and thus grabbing the handlebar), driving a car (and thus grabbing the steering wheel), carrying shopping bags in your hands, or paddling in a canoe involve a contraction of the forearm muscles to grasp an object with your hands; therefore, they can generate similar electromyographic recordings. However, presenting a different electromyographic activity in the forearms provides strong evidence that a different action is being performed: if the electromyographic activity indicates that there is no contraction in the forearms, it is possible to rule out that the subject is performing any of the four activities previously described (negative evidence).

This situation is usually addressed in ensemble clustering by discarding these features in the data representation. Nevertheless, there are scenarios in which the information of the elements belonging to different clusters can be valuable in order not to group those elements together in the final data representation. We have named this evidence as *negative evidence*. The data partitions that are used to extract negative evidence will be referred to as $E^{-}=\left\{P_{1}^{-},P_{2}^{-},\ldots ,P_{o}^{-}\right\}$, $E^{-}\subset E$, where *o* is the number of negative evidence partitions, to distinguish them from the partitions used to extract positive evidence. It must be noted that these *o* data partitions correspond to a subset of the original *m* data partitions, and that $E=E^{+}\cup E^{-}$.

To gather this negative evidence, we also make use of a voting mechanism and a matrix. The difference is that, in this case, for each partition in which the elements *i* and *j* are not in the same cluster, we will compute a negative vote in the corresponding cell of the matrix. The negative evidence matrix $G^{-}$, whose size is n×n, will be:

(3) $$G_{\left(i,j\right)}^{-}=-\frac{o_{ij}}{o},$$

where $o_{ij}$ is the number of times that the pair of elements *i* and *j* has been assigned to different clusters in the different data partitions $P_{1}^{-},P_{2}^{-},\ldots ,P_{o}^{-}$.

It is important to remark that the data partitions employed for extracting the negative evidence cannot be the same as those used for extracting the positive evidence. That is, each partition can only provide one type of evidence. Moreover, each type of evidence should be generated using suitable data representations. It is also important to note that negative evidence can only be used altogether with positive evidence. Negative evidence provides information about those elements that should not be grouped together, behaving like a sort of restrictions, but this information is only useful when jointly used with the information about which elements should be grouped together (obtained from the positive evidence).

After extracting the positive evidence and, when applicable, the negative evidence, the PN-EAC algorithm combines both evidence matrices in a single one to obtain the final evidence matrix $G^{*}$. This can be done by adding all the different matrices, as follows:

(4) $$G^{*}=G^{+}+G^{-}.$$

This simple combination of multiple data partitions supports the integration of different sources of information in parallel. Therefore, PN-EAC will be able to combine information from different sensors. The algorithm is versatile, allowing the customization of the ensemble scheme combining positive and negative evidence, using different algorithms to generate the partitions or changing the number of partitions.

### 2.3. Obtaining the Final Data Partition

The last step of PN-EAC is obtaining the final data partition $P_{*}$ using the evidence matrix $G^{*}$, which behaves like a similarity matrix between all the elements. Any clustering algorithm that accepts as input a similarity matrix could be used to obtain the final partition. In the PN-EAC, the use of a hierarchical linking clustering algorithm [34] is proposed for this purpose. This algorithm is designed to be applied to similarity matrices and, therefore, is suited to be applied to the evidence matrix $G^{*}$ [35].

This hierarchical clustering algorithm establishes a hierarchy of clusters, which are organised into a tree-like structure called a dendrogram [36]. To create this structure, two different strategies can be used: agglomerative or divisive. In the agglomerative strategy, which is the one used in the PN-EAC, each element starts as a cluster and, in each iteration, two clusters are merged. In the divisive strategy, there is only one cluster at the beginning and in each iteration one cluster is split in two. To merge or split the clusters, a measure of distance between elements is needed. In the PN-EAC, the Euclidean distance is used. On the other hand, it is also necessary to decide the method to calculate the distance between two clusters; typical choices include using the mean of all the distances between their elements (average link), using only the longest distance (complete link) or using the smallest distance (simple link), among others [37]. The average link [38] was the choice for the PN-EAC. Therefore, the final partition $P_{*}$ is obtained using a hierarchical linking clustering algorithm based on the Euclidean distance over the evidence matrix $G^{*}$. The results of the hierarchical algorithm are typically presented using a dendrogram, which is a tree-like diagram that shows the different clusters, their similarity values and how they are joined and split in the different iterations.

**Figure 2:**
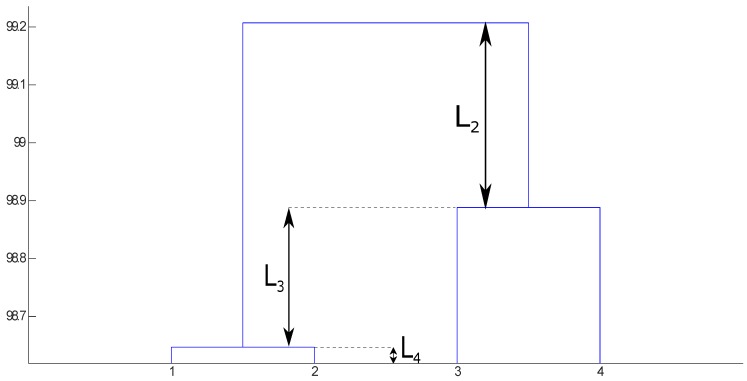
Example of a dendrogram illustrating the lifetime criterion. The original data are represented as the leaves of the tree (bottom part of the figure) and with different *x*-values. Every time two groups are merged, they are joined in a branch of the tree. In the *y*-axis, the height of the branch is the so-called lifetime, which represents the dissimilarity between groups. The figure represents graphically the lifetime values for 2 ($L_{2}=0.31$), 3 ($L_{3}=0.23$) and 4 ($L_{4}=0.02$) clusters. The final number of clusters according to the lifetime criterion is 2, since $L_{2}$ is the largest value.

Most clustering ensemble algorithms leave the user with the decision concerning the final number of clusters. To that end, the user typically decides to “cut” the dendrogram at some point to generate a certain number of clusters. In [31], an alternative is proposed: the use of a criterion based on the lifetime of each cluster in the dendrogram. It is possible to define the lifetime of a number of clusters *k* as the absolute similarity difference in the dendrogram between the transition of k+1 to *k* clusters and the transition between *k* to k−1 clusters (see Figure 2). This process is repeated for all the possible values of *k*. The *k* with the largest value is chosen as the final number of clusters. Figure 2 shows an illustrative example: the lifetime values for two, three, and four clusters are shown, named $L_{2}$, $L_{3}$ and $L_{4}$, respectively; the largest value is $L_{2}$ and therefore k=2 is chosen as the number of clusters. This criterion is based on the idea that the most similar partition to the “natural” partition of the data will be more stable in regard to merging or splitting clusters in the dendrogram; in other words, it will have the longest lifetime.

### 2.4. Usefulness of the Negative Evidence: An Illustrative Example

**Figure 3:**
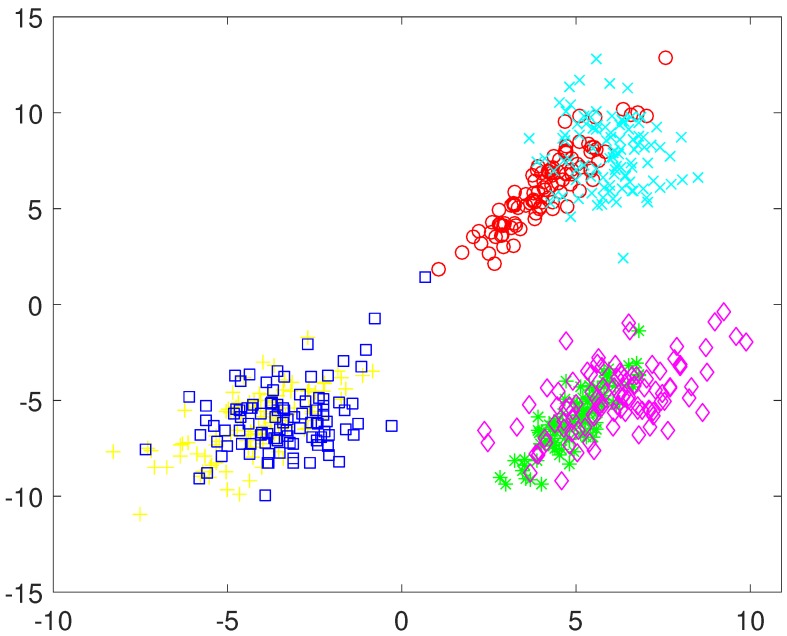
Representation of the first two features of the example data set.

**Figure 4:**
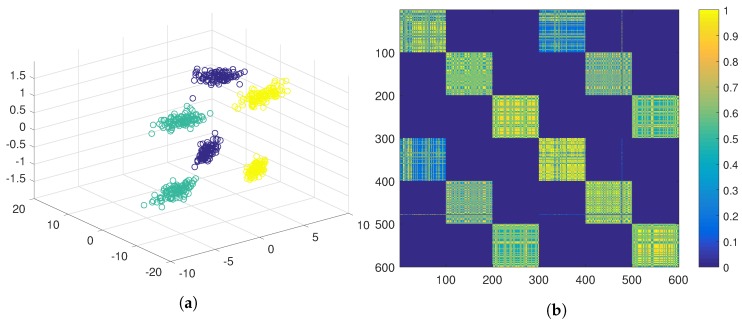
PN-EAC extracting positive evidence with the three features in the same feature vector. (**a**) Cluster result in 3D; (**b**) Representation of the similarity matrix.

To illustrate the use of the negative evidence in the PN-EAC algorithm, a data set composed of 600 elements from six different Gaussian distributions was built. The data set has three features. The first two features are represented in Figure 3, and the last feature is a Gaussian with mean of −1 for three clusters and another Gaussian with mean 1 for the other three clusters. A three-dimensional representation of the data can be seen in Figure 4a.

Using the traditional ensemble clustering, positive evidence would be extracted using the three features joined up in the same feature vector, and gathered in the evidence matrix, $G^{*}=G^{+}$ (see Equation (4)). This evidence matrix is displayed as a similarity matrix in Figure 4b using a colour code, where yellow represents high similarity and blue represents low similarity. The similarity matrix has been ordered according to the ground true labels of the six clusters. In this similarity matrix, it can be observed that, although the elements belonging to the same natural cluster are very similar to the rest of the elements of the same cluster, they are also similar to the elements of a second cluster. When hierarchical clustering is applied over this matrix, the six natural clusters are grouped in pairs (see Figure 4a), hence obtaining three clusters.

**Figure 5:**
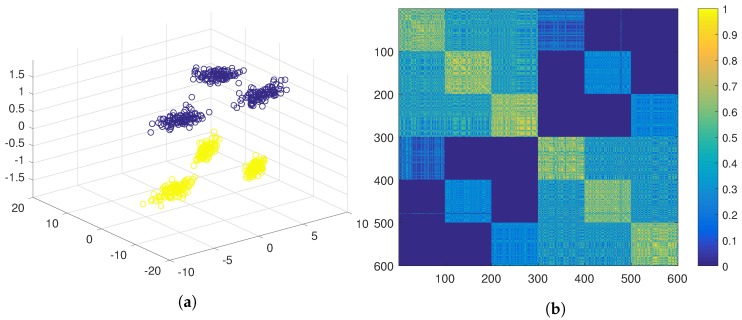
PN-EAC extracting and combining positive evidence separately from two sources: the first two features and the last one. (**a**) Cluster result in 3D; (**b**) Representation of the similarity matrix.

**Figure 6:**
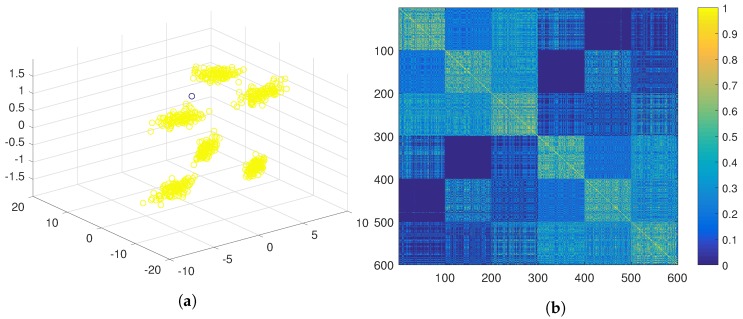
PN-EAC extracting and combining positive evidence from the three features separately. (**a**) Cluster result in 3D; (**b**) Representation of the similarity matrix.

A different approach would be obtaining positive evidence separately from the first two features and from the third one independently. This approach yields a much less crisp similarity matrix (see Figure 5b), which results in two clusters after the application of the hierarchical algorithm (see Figure 5a). Another option would be to obtain positive evidence separately from each of the three features. The similarity matrix obtained in this case is even less crisp than in the previous case (see Figure 6b), which results in a cluster containing almost all the samples (see Figure 6a).

At this point, the data analyst might think that the third feature is of poor quality and/or it introduces too much noise, and it could be a good solution to discard it. However, it is clear from Figure 3 that, if the third feature is discarded, it is not possible to separate the six natural clusters from the two remaining features.

**Figure 7:**
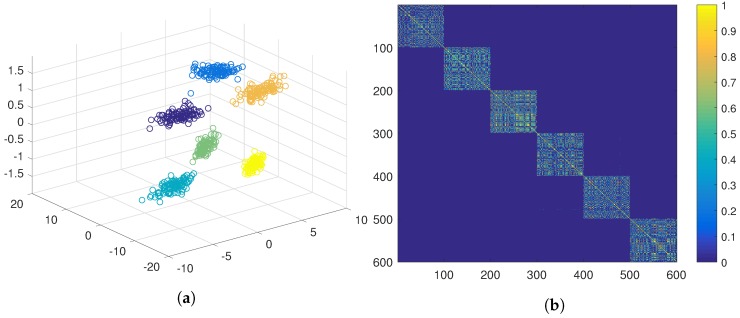
PN-EAC extracting and combining evidence from two sources: positive evidence from the first two features and negative evidence from the last feature. (**a**) Cluster result in 3D; (**b**) Representation of the similarity matrix.

Figure 4, Figure 5 and Figure 6 show strategies framed in the classic evidence accumulation cluster paradigm. We will make a third attempt with the PN-EAC algorithm, proceeding in a similar way to the strategy of Figure 5, but, in this case, the third feature is used to extract negative evidence ($G^{-}$, see Equation (3)), which is combined with the positive evidence ($G^{+}$, see Equation (2)) extracted from the two other features $G^{*}=G^{+}+G^{-}$ (see Equation (4)). Figure 7b shows the similarity matrix resulting from this approach, and Figure 7a shows the clusters obtained after the application of the hierarchical clustering. Note how, by using the third feature to extract information about which elements should not be grouped together, we get a more crisp similarity matrix that reflects more adequately the natural partition underlying the data set than any of the other two previous evidence matrices. At this point, we would like to emphasize that the only difference between the results presented in Figure 5 and in Figure 7 is the change in the type of evidence extracted from the third characteristic: positive in the first case and negative in the second one.

This is a simple toy example designed to illustrate how negative evidence can be useful to address some situations that it is not possible to solve satisfactorily in a paradigm that only contemplates positive evidence. As a general rule, any feature in which similarity means belonging to the same cluster should be used to extract positive evidence. If the feature does not fulfil this condition, it may be considered to extract negative evidence. To extract meaningful negative evidence, dissimilarity in that feature should be useful to distinguish between some clusters.

### 2.5. Computational Complexity of PN-EAC

To study the computation complexity of PN-EAC, a complexity analysis of each of its component parts is performed. The complexity of the K-means algorithm used to generate the partitions (see Algorithm 1, line 1) has been widely studied in the bibliography [39]: O(n·k·d·i), where *n* is the number of elements, *k* is the number of clusters, *d* is the number of dimensions of each feature vector, and *i* is the number of iterations of the algorithm. The number of dimensions *d* is usually fixed for almost any problem and is typically much smaller than *n*. The same occurs with the number of iterations *i*, which is often set to a fixed amount (typically 100). The number of clusters in our algorithm is given by Equation (1) with a maximum of $\sqrt{n}$. Therefore, the complexity of K-means can be approximated by $O\left(n\cdot k\cdot d\cdot i\right)\approx O\left(n\cdot \sqrt{n}\right)$. The K-means algorithm is executed once per data partition (see Algorithm 1, line 1). Thus, the complexity of this step of the algorithm is $O(m\cdot n\cdot \sqrt{n})$, where *m* is the number of data partitions.

Extracting evidence from the partitions requires to iterate through the evidence matrix, thus the complexity is determined by the size of the matrix (n×n), being $O(n^{2})$. The matrix is processed once per partition, so the final computational complexity of this step will be $O(m\cdot n^{2})$ (see Algorithm 1, line 2).

Finally, it is necessary to consider the complexity of the hierarchical algorithm used to extract the final partition. In the algorithm, the first step is to calculate the similarity matrix between all the elements; this operation has a complexity of $O(n^{2})$. Afterwards, *n* iterations will be executed, one per element, and, in each iteration, the pair of clusters with the smallest distance will be merged and the distance to the other clusters will be updated. The complexity of each iteration is O(n·log(n)). Given that there are *n* iterations, the total complexity will be $O(n^{2}\cdot log\left(n\right))$, this term being the one that determines the complexity of the hierarchical algorithm [36] (see Algorithm 1, line 3).

The complexity of the hierarchical algorithm ($O(n^{2}\cdot log\left(n\right))$) can be larger than the complexity of extracting evidence ($O(m\cdot n^{2})$), in the case of log(n) is larger than *m*. However, for typical values of *n* and *m*, m>>log(n) stands. For example, with 300 different data partitions (*m*), *n* is almost always smaller than $2^{300}$. Thus, the complexity of the PN-EAC algorithm will be $O(m\cdot n^{2})$, as shown next:

(5) $$O\left(m\cdot n\cdot \sqrt{n}\right)+O\left(m\cdot n^{2}\right)+O\left(n^{2}\cdot log\left(n\right)\right)\approx O\left(m\cdot n^{2}\right).$$

Regarding the space complexity, it will be rendered by the size of the evidence matrix, $O(n^{2})$. In an optimized representation, it could be reduced to $O(n^{2}/2)$, given that the matrix is symmetric.

### 2.6. PN-EAC for Heartbeat Clustering

One of the main difficulties in heartbeat clustering lies in the morphological complexity of the heartbeat. The inherent variability of any morphological family of heartbeats, namely, the group of heartbeats that shares origin of activation and propagation path, is not projected in the feature space in hyperspherical shape. No matter the heartbeat representation chosen, it has been shown that the different morphological families have a heterogeneous set of shapes in the feature space [40].

To represent the morphology of the heartbeat, a decomposition based on the Hermite functions shall be used. This representation is robust and compact due to the similarity between the shape of the Hermite functions and the QRS complexes. Furthermore, the Hermite functions are orthonormal; thus, each feature provides independent information. An excerpt of 200 ms is extracted for each heartbeat around the beat position. By means of this window size, the complete QRS complex is extracted, whereas the P and T waves are ignored. The number of Hermite functions used in the decomposition is 16 (plus a σ parameter to control the width of the QRS complex), since this value provides a good compromise between the length of the feature vector and the accuracy of the heartbeat representation [41].

**Figure 8:**
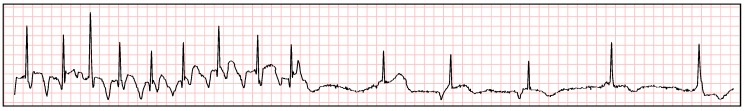
Electrocardiogram (ECG) fragment with several normal beats at the beginning. Around the middle of the fragment, the distances between the heartbeats increase and escape beats appear. (Source: MIT-BIH Arrhythmia Database, recording 222, from 0:12:36 to 0:12:46).

**Figure 9:**
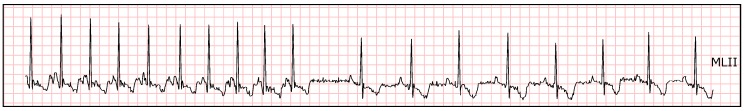
Electrocardiogram (ECG) fragment with Nodal premature beats (J) at the beginning. Around the middle of the fragment, the distance between heartbeats increases and normal beats appear. (Source: MIT-BIH Arrhythmia Database, recording 234, from 0:14:27 to 0:14:37).

There are some types of arrhythmia, like premature atrial contractions and atrioventricular, or fusion and escape beats, in which the morphology of the QRS complexes is similar. Therefore, it is not feasible to distinguish between them using just QRS morphology information (see Figure 8 and Figure 9). The P wave does provide relevant information in these cases, but the difficulty in identifying it, especially in ambulatory ECG, often leads to attempts to obtain similar information from the distance between heartbeats [13,42]. Therefore, to allow the clustering algorithm to distinguish between different types of heartbeats with similar QRS morphology, two features based on the distance between heartbeats ($R_{1}$ and $R_{2}$) were added as follows:

(6) $$R_{1}\left[i\right]=R\left[i\right]-R\left[i-1\right],$$

(7) $$\begin{array}{c} \alpha \left[i\right]=\left(R_{1}\left[i+1\right]-R_{1}\left[i\right]\right)-\left(R_{1}\left[i\right]-R_{1}\left[i-1\right]\right),\\ R_{2}\left[i\right]=u\left(\alpha \left[i\right]\right)\cdot \alpha \left[i\right], \end{array}$$

where R[i] is the instant of time of the occurrence of the *i*-th heartbeat, and α[i] is the difference between the distance from the *i*-th beat to the next one, and the distance from the *i*-th beat to the previous one. The Heaviside step function u(x) is:

(8) $$u\left(x\right)=\{\begin{array}{ccc} 1, & & \mathrm{if}\;x\geq 0,\\ 0, & & \mathrm{if}\;x<0. \end{array}$$

This function is used to remove the negative values of α[i] in Equation (7) (clipping them to 0) while preserving the positive values. Equation (6) measures the distance from a heartbeat to the previous one. By comparing the values of this equation, it is possible to identify if a heartbeat is premature. To this effect, Equation (7) compares this distance with other two distances: the distance between the current beat and the next one, and the distance between the previous beat and its predecessor. A high α[i] value in Equation (7) provides evidence that the heartbeat could be premature. Thus, each heartbeat is represented by the coefficients of the Hermite functions for each of the different available leads, the σ parameters and the features given by Equations (6) and (7).

#### Strategies for Generating Partitions

When applying the PN-EAC algorithm to heartbeat clustering, three different strategies are proposed to generate the data partitions. These three strategies have been designed to assess the value of the negative evidence, as well as to study the performance of the algorithm when integrating more data sources incrementally.

**Figure 10:**
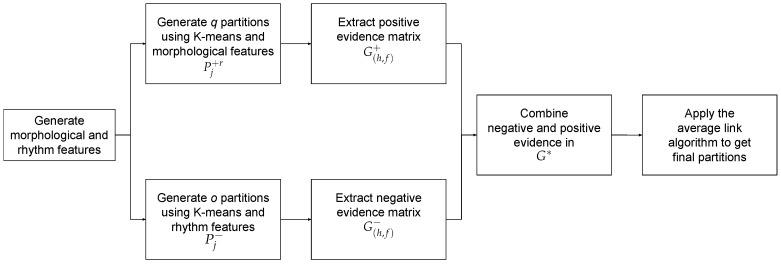
Block diagram summarizing the main steps of strategy 3 of PN-EAC.

**Figure 11:**
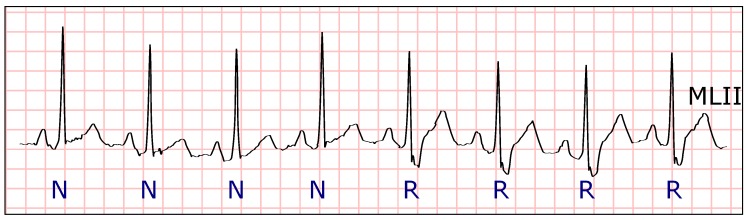
A fragment of an electrocardiogram (ECG) with four normal heartbeats (**N**) followed by four heartbeats with right branch block (**R**). It is important to emphasize that the distance between heartbeats is similar for the whole fragment. Therefore, the two different types of heartbeats have very similar values in Equations (6) and (7). (Source: MIT-BIH Arrhythmia Database, recording 212, from 0:12:13 to 0:12:18).

**Figure 12:**
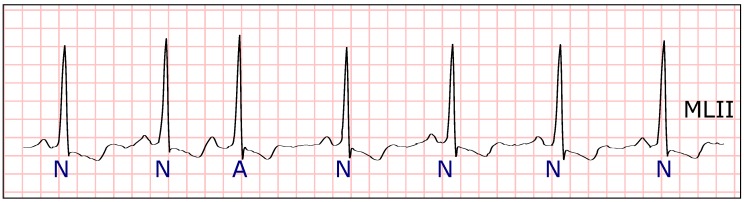
In this fragment of electrocardiogram (ECG), the third heartbeat is a premature atrial beat (**A**), while the others are normal heartbeats (**N**). It is possible to note that the abnormal beat is morphologically similar to the normal beats, but the distance with the surrounding heartbeats changes. (Source: MIT-BIH Arrhythmia Database, recording 223, from 0:01:02 to 0:01:07).

**Strategy** **1** The first strategy is the classical approach of machine learning whereby all the available features of an element are grouped into a single feature vector. In this work, the vector has the parameters from the Hermite representation (coefficients and σ) for all the ECG leads and the features given by Equations (6) and (7), as follows:
  (9) $$u_{i}=\left(C^{1},\sigma^{1},C^{2},\sigma^{2},\ldots ,C^{d},\sigma^{d},R_{1},R_{2}\right),$$
  where $u_{i}$ is the feature vector that represents the heartbeat $x_{i}$; *d* is the number of leads, $C^{j}$ denotes the Hermite coefficients that represent the QRS complex over the lead *j*, $C^{j}=\left\{c_{0}\left(\sigma^{j}\right),c_{1}\left(\sigma^{j}\right),\ldots ,c_{N-1}\left(\sigma^{j}\right)\right\}$ (in this case with N=16, i.e., the number of Hermite coefficients); and $\sigma^{j}$ is the value of σ in the lead *j*. Each partition uses the representation from Equation (9) for each heartbeat, with which the positive evidence is extracted. To create the different partitions $P_{1}^{+},P_{2}^{+},\ldots ,P_{q}^{+}$, the K-means algorithm will be used with random initializations and a number of clusters in the range given by Equation (1) (see Algorithm 2, lines 3–6).
**Strategy** **2** The second strategy is based on the hypothesis that generating the data partitions from different data representations yields better results than integrating all the features in the same feature vector. This strategy is inspired by cardiologists, who typically study each derivation separately and then combine the findings on each of them to make the final interpretation. This strategy mimics this *modus operandi*, extracting evidence from the information of each lead and combining it for a final decision. The information of the distance between the heartbeats given by Equations (6) and (7) is also processed separately. This is due to the fact that it is a different type of information compared to the morphology information. Thus, the features are divided in different representations of the heartbeats: *d* feature vectors for the morphological information of all leads (represented by the Hermite functions) and another feature vector with the information derived from the distance between heartbeats, as follows:
  vi1=(C1,σ1),vi2=(C2,σ2),…,(10)vid=(Cd,σd),
  (11)wi=(R1,R2),
  where $v_{i}^{1}$, $v_{i}^{2}$, …, $v_{i}^{d}$ are the feature vectors of the different leads representing the heartbeat $x_{i}$ by means of the Hermite representation, *d* is the number of leads and $w_{i}$ is the representation of $x_{i}$ based on the distance between heartbeats. Each representation is used separately to generate the data partitions to extract positive evidence. The final data partition is obtained by combining all the evidence (see Algorithm 3, lines 25–26).
**Strategy** **3** The third strategy is a variant of the second one, but, in this case, negative evidence is extracted using the features derived from the distances between heartbeats ($w_{i}$). This strategy is summarized in Figure 10. The fact that the values of Equations (6) and (7) are not different, (i.e., the distance between heartbeats does not change) does not imply that there is not a change in the type of heartbeats; there is a wide variety of types of heartbeats that have a similar distance between beats. Thus, similar values for those features do not provide conclusive evidence whether two heartbeats belong to the same cluster (see Figure 11). However, two heartbeats with remarkable differences in those values are very likely to belong to different types (see Figure 12).
  In this strategy, to model the information obtained from Equations (6) and (7), negative evidence will be used. To represent the heartbeats, the same representations that in the previous strategy are used (see Equations (10) and (11)). However, in this case, negative evidence is extracted from Equation (11). The other representations are still used to generate positive evidence (see Algorithm 4, lines 21–25).

**Algorithm 2** Heartbeat Clustering with PN-EAC (Strategy 1).**Require:** Set of heartbeats $X=\{x_{1},x_{2},\ldots ,x_{n}\}$, number of partitions *q***Ensure:**$P_{*}$ final data partition of the *n* elements
1:**for all** heartbeat $x_{i}$ **generate** $u_{i}$ using Equation (9)

2:
$U=\{u_{1},u_{2},\ldots ,u_{n}\}$


3:**for***j* = 1 *q* **do**
#Section 2.1
4: $k=random(\left[\sqrt{n}/2,\sqrt{n}\right])$
#Equation (1)
5: $P_{j}^{+}=K\text{-}means\left(U,k\right)$


6:
**end for**


7:
$G^{+}=zeros\left(n,n\right)$

#Initialize positive evidence matrix
8:**for***j* = 1 *q* **do**
#For each partition (see Section 2.2)
9: **if** two heartbeats $u_{h}$ and $u_{f}$ are in the same cluster in $P_{j}^{+}$ **then**

10:  $G_{\left(h,f\right)}^{+}=G_{\left(h,f\right)}^{+}+1$
#Equation (2); extract positive evidence
11: **end if**

12:
**end for**


13:
$G^{*}=G^{+}/q$

#Equation (2)
14:
$P_{*}=average.link\left(G^{*}\right)$

#Section 2.3

**Algorithm 3** Heartbeat Clustering with PN-EAC (Strategy 2).**Require:** Set of heartbeats $X=\{x_{1},x_{2},\ldots ,x_{n}\}$**Require:** Number of partitions for each lead (*q*) and for the distances between beats (*b*)**Ensure:**$P_{*}$ final data partition of the *n* elements
1:**for all** heartbeat $x_{i}$ **generate** $v_{i}^{1}$; $v_{i}^{2}$; … $v_{i}^{d}$; $w_{i}$ using Equations (10) and (11)

2:**for***r* = 1 *d* **do**
#For each lead
3: $Vr=\{v_{1}^{r},v_{2}^{r},\ldots ,v_{n}^{r}\}$

4: **for** *j* = 1 *q* **do**            #Section 2.1; *q* partitions generated with morphological information

5:  $P_{j}^{+r}=K\text{-}means\left(Vr,k=random\left(\left[\sqrt{n}/2,\sqrt{n}\right]\right)\right)$


6: **end for**

7:
**end for**


8:
$W=\{w_{1},w_{2},\ldots ,w_{n}\}$


9:**for***j* = 1 *b* **do**                   #Section 2.1; *b* partitions generated from Equation (11)

10: $P_{j}^{+w}=K\text{-}means\left(W,k=random\left(\left[\sqrt{n}/2,\sqrt{n}\right]\right)\right)$


11:
**end for**


12:
$G^{+}=zeros\left(n,n\right)$

#Initialize positive evidence matrix
13:**for***j* = 1 *q* **do**        #For each partition obtained from morphological information (see Section 2.2)

14: **for** *r* = 1 *d* **do**
#For each lead
15:  **if** the representations of two heartbeats $v_{h}^{r}$ and $v_{f}^{r}$ are in the same cluster in $P_{j}^{+r}$ **then**

16:   $G_{\left(h,f\right)}^{+}=G_{\left(h,f\right)}^{+}+1$
        #Equation (2); extract positive evidence
17:  **end if**

18: **end for**

19:
**end for**


20:**for***j* = 1 *b* **do**        #For each partition obtained from the distance between beats (see Section 2.2)

21: **if** the representations of two heartbeats $w_{h}$ and $w_{f}$ are in the same cluster in $P_{j}^{+w}$ **then**

22:   $G_{\left(h,f\right)}^{+}=G_{\left(h,f\right)}^{+}+1$
 #Equation (2); extract positive evidence
23: **end if**

24:
**end for**


25:
$G^{*}=G^{+}/\left(\left(d\cdot q\right)+b\right)$

#Equation (2)
26:
$P_{*}=average.link\left(G^{*}\right)$

#Section 2.3

**Algorithm 4** Heartbeat Clustering with PN-EAC (Strategy 3).**Require:** Set of heartbeats $X=\{x_{1},x_{2},\ldots ,x_{n}\}$, number of positive partitions for each lead (*q*)**Require:** Number of negative partitions for the distances between beats (*o*)**Ensure:**$P_{*}$ final data partition of the *n* elements
1:**for all** heartbeat $x_{i}$ **generate** $v_{i}^{1}$; $v_{i}^{2}$; …; $v_{i}^{d}$; $w_{i}$ using Equations (10) and (11)

2:**for***r* = 1 *d* **do**
#For each lead
3: $Vr=\{v_{1}^{r},v_{2}^{r},\ldots ,v_{n}^{r}\}$


4: **for** *j* = 1 *q* **do**            #Section 2.1; *q* partitions generated with morphological information

5:  $P_{j}^{+r}=K\text{-}means\left(Vr,k=random\left(\left[\sqrt{n}/2,\sqrt{n}\right]\right)\right)$


6: **end for**

7:
**end for**


8:
$W=\{w_{1},w_{2},\ldots ,w_{n}\}$


9:**for***j* = 1 *o* **do**                   #Section 2.1; *o* partitions generated from Equation (11)

10: $P_{j}^{-}=K\text{-}means\left(W,k=random\left(\left[\sqrt{n}/2,\sqrt{n}\right]\right)\right)$


11:
**end for**


12:
$G^{+}=zeros\left(n,n\right)$

#Initialize positive evidence matrix
13:
$G^{-}=zeros\left(n,n\right)$

#Initialize negative evidence matrix
14:**for***j* = 1 *q* **do**        #For each partition obtained from morphological information (see Section 2.2)

15: **for** *r* = 1 *d* **do**
#For each lead
16:  **if** the representations of two heartbeats $v_{h}^{r}$ and $v_{f}^{r}$ are in the same cluster in $P_{j}^{+r}$ **then**

17:   $G_{\left(h,f\right)}^{+}=G_{\left(h,f\right)}^{+}+1$
#Equation (2); extract positive evidence
18:  **end if**

19: **end for**

20:
**end for**


21:**for***j* = 1 *o* **do**       #For each **negative** evidence partition obtained from the distance between beats

22: **if** the representations of two heartbeats $w_{h}$ and $w_{f}$ are **not** in the same cluster in $P_{j}^{-}$ **then**

23:   $G_{\left(h,f\right)}^{-}=G_{\left(h,f\right)}^{-}-1$
#Equation (3); extract negative evidence
24: **end if**

25:
**end for**


26:
$G^{*}=G^{+}/\left(d\cdot q\right)+G^{-}/o$

#Equations (2), (3) and (4)
27:
$P_{*}=average.link\left(G^{*}\right)$

 #Section 2.3

## 3. Experimental Setup

The code needed for the PN-EAC algorithm was developed using Matlab (R2015b). The implementations of K-means and the agglomerative hierarchical clustering algorithm (linkage) were taken from the Statistics and Machine Learning Toolbox. Experiments were run on a laptop with an i7 dual core processor with 16 GB of RAM. To validate the PN-EAC algorithm, the annotations marking the position and the type of each heartbeat in the MIT-BIH Arrhythmia and INCARTDB databases were used as ground-truth. To evaluate the accuracy of the clustering algorithm, we considered that a cluster belongs to the type of the majority of its heartbeats, according to the annotations in the database. All the heartbeats in the cluster that are from a different type compared to that of the ground-truth are considered errors. In the clinical routine, if necessary, the correspondence between clusters and heartbeat types could be obtained by having a cardiologist annotating one beat of the majority morphology in each cluster.

The following subsections briefly introduce the databases used along with the experimental methods employed in each database.

### 3.1. MIT-BIH Arrhythmia Database

The MIT-BIH Arrhythmia Database is the most referenced ECG database [43]. The wide variety of patients, the different types of heartbeats and the quantity and quality of the annotations have made this database a “gold-standard” [12,42,44,45,46,47,48]. It is composed of 48 recordings from 47 different patients. Each recording has two leads among MLII, V1, V2, V3, V4 and V5. The recordings are digitized at 360 Hz, with around 30 min each. They consist of normal heartbeats (68% of the data) and 16 types of arrhythmia, for which the corresponding labels are also included.

In this paper, the MIT-BIH Arrhythmia Database is completely processed, without discarding any recording. Each heartbeat is represented by 36 features, the 16 coefficients of Hermite representation per lead, the value of σ per lead and the two features based on the distance between heartbeats, given by Equations (6) and (7). To generate the data partitions, the K-means algorithm is employed, using different initializations and a random number of clusters in the range given by Equation (1).

In the first strategy, all the features are in the same feature vector $u_{i}$ and 300 different data partitions are generated for each recording. Likewise, 100 partitions for the second and third strategies are generated for each of the feature vectors ($v_{i}^{1}$, $v_{i}^{2}$ and $w_{i}$), yielding a total of 300 partitions for each strategy. In the case of the second strategy, positive evidence is extracted from the three feature vectors, while in the case of the third one, positive evidence is extracted from the feature vectors corresponding to the morphology of the heartbeat ($v_{i}^{1}$ and $v_{i}^{2}$) and negative evidence is extracted from the feature vector corresponding to the distance between beats ($w_{i}$). The final data partition $P_{*}$ is extracted from the evidence matrix applying the average link algorithm (see Algorithm 2, line 14; Algorithm 3, line 26; Algorithm 4, line 27).

### 3.2. St. Petersburg Institute of Cardiological Technics 12-Lead Arrhythmia Database

In the clinical routine, the ECG is commonly registered using 12 leads, and all of them are used by the cardiologist for the diagnostic [49]. However, in the literature of heartbeat classification and clustering, only one or two leads of the ECG recordings are typically employed [12,42,45]. One of the main reasons for this is that the reference database for arrhythmia identification, the MIT-BIH Arrhythmia Database, has only two leads. Notwithstanding, there are other annotated databases with more leads, like the St. Petersburg Institute of Cardiological Technics 12-Lead Arrhythmia Database [50]. This database has 75 recordings sampled at 257 Hz and obtained from 32 different patients. The variety of cardiac problems is not as wide as in the MIT-BIH Arrhythmia Database, but in exchange it has a larger number of recordings and more leads per recording. It is one of the few databases with 12 leads with beat by beat annotations and is freely available. Therefore, its use is extended [51,52,53]. Each recording is an extract of 30 min and most of them have the 12 standard leads.

In the tests with the INCARTDB, we only use 71 of the 75 recordings. Recordings I02, I03, I57 and I58 were excluded since at least one of the leads is missing. By applying PN-EAC to the INCARTDB, it is possible to study the performance of the algorithm using up to 12 leads. To that end, we apply the third strategy by increasing the number of leads from 2 to 12 in a 2-wise step. This test will allow us to see how the performance of the PN-EAC algorithm behaves when it is used to merge a growing number of data sources.

For each lead, the Hermite representation will be obtained (coefficients and σ parameter), using 16 functions. Afterwards, the features derived from the distance between heartbeats (see Equations (6) and (7)) will be calculated, using the annotations of the database. Consequently, each heartbeat will be characterized by up to twelve representations of Hermite (16 coefficients and σ), one per lead, and the two features derived from the distance between heartbeats. This adds up to 206 features per heartbeat. This dimensionality in the feature space could be too high for most machine learning algorithms, requiring the use of techniques for feature selection and/or techniques to reduce the number of dimensions [54].

To apply the algorithm with two leads, the procedure is analogous to the one presented previously for the MIT-BIH Arrhythmia Database. For each of the two leads, 100 data partitions are generated to extract positive evidence. These partitions, like all the other partitions, were generated using the K-means algorithm. In the INCARTDB, it is necessary to choose two leads from the 12 leads available. This selection was performed randomly. In addition, 100 partitions were generated using the features given by Equations (6) and (7), and negative evidence will be extracted from these partitions. The whole process will be repeated 100 times to obtain the mean error of using two random leads.

To apply PN-EAC with four leads, the process will be similar. The first step is to randomly select 4 out of the 12 leads available. For each of these leads, 100 data partitions will be generated using the Hermite information (coefficients and σ). Consequently, there are 400 partitions in total to extract positive evidence. To maintain the same relative weight of the positive evidence regarding the negative evidence used previously (i.e., 1/3 of the total evidence), 200 data partitions used to extract negative evidence will be generated from the features derived from the distance between heartbeats given by Equations (6) and (7). Avoiding the dilution of this information is important since the information derived from the Hermite representation and the information of the distance between heartbeats are complementary. For the cases of 6, 8, 10 and 12 leads, the procedure is equivalent, obtaining 100 partitions for each lead to generate positive evidence and adjusting the number of partitions generated with the features given by Equations (6) and (7), so the number of partitions that provide negative evidence is always 1/3 of the total number of partitions.

For each number of leads, the whole process is repeated 100 times. In each iteration, the leads are randomly chosen between all the 12 available leads, except in the case of 12 leads where all leads are used. The generation of the data partitions and the extraction of the final partition from the evidence matrix is carried out using Algorithm 4.

## 4. Results

This section presents the results obtained with the PN-EAC on the two databases (MIT-BIH Arrhythmia Database and INCARTDB).

### 4.1. MIT-BIH Arrhythmia Database

The results per recording for the three strategies are shown in Table 1. For each recording, the number of errors using a fixed number of 25 clusters per recording (25C) and the number of clusters and errors when using the lifetime criterion (Lifetime) are specified. The total number of errors and the percentage of the whole database that it represents (109,966 heartbeats) also appear at the end of Table 1. Using a fixed number of 25 clusters per recording, percentages of error of 2.25%, 1.81% and 1.44% are obtained for the first, second and third strategies, respectively. Using the lifetime criterion the error percentages are 2.75%, 3.81% and 0.99% with a total number of 508, 203 and 6947 clusters for the first, second and third strategies, respectively.

Statistical tests were applied to the results of Table 1 to verify if the differences between strategies were significant or not. It was found that the variables do not follow normality using the Shapiro–Wilk test [55]. Thus, the non-parametric Wilcoxon test [56] was applied to the results of the different strategies. For the fixed number of 25 clusters, we obtain *p*-values of 0.0821, <0.0001 and 0.0404 for Strategy 1 vs. Strategy 2, Strategy 1 vs. Strategy 3 and Strategy 2 vs. Strategy 3, respectively (see Table 2). With a significance level of 0.05, it is not proved that the second strategy is better than the first one; however, the third strategy, which uses negative evidence, is significantly better than the first and the second strategies. For the lifetime criterion results, the *p*-values were <0.01 for all the cases (see Table 2), and therefore all the results are significantly different with this criterion.

The confusion matrix for the third (the best) strategy and 25 clusters was calculated. The matrix was obtained using both the original annotations from the MIT-BIH Arrhythmia Database (see Table 3) and the annotations recommended by the Association for the Advancement of Medical Instrumentation (AAMI) (see Table 4). In these matrices, the columns represent the real type of heartbeat according to the annotations and the rows represent the type assigned by the clustering algorithm to the corresponding heartbeat.

### 4.2. INCARTDB

Results corresponding to the INCARTDB are shown in Table 5, in which the average error obtained with the third strategy and 25 clusters for the different numbers of leads is presented. In this case, the lifetime criterion is not used due to the bad performance obtained with the PN-EAC, especially regarding the proliferation of clusters. We used the third strategy because, as shown in the results obtained on the MIT-BIH Arrhythmia Database, is the one that obtained the best performance. Therefore, the results are always obtained using a fixed number of 25 clusters, using the third strategy (see Algorithm 4) and varying the number of leads used to generate evidence. The percentage of error is obtained dividing the total number of errors by the total number of heartbeats in the database (165,514 heartbeats, around 2300 per recording). The mean error percentages for 100 executions are 0.6006%, 0.4052%, 0.381%, 0.3579%, 0.3493% and 0.3379% for 2, 4, 6, 8, 10 and 12 leads, respectively.

Statistical tests were applied on the results to check if the differences from using a different number of leads were significant [55]. The normality hypothesis was rejected and thus non-parametric tests were applied. First, the Friedman test was applied [57]. It takes as a null hypothesis that the results obtained with different number of leads are all equal. This hypothesis was rejected (*p*-value < 0.001), proving therefore that there are significant differences in the results obtained using different numbers of leads. Additionally, the different options were compared in pairs to check if there were differences when adding more leads. For that end, the Wilcoxon test [56] was used to carry out pairwise comparisons. In all the cases, the *p*-value obtained was smaller than 0.001, hence always rejecting the null hypothesis. Therefore, there are significant differences in all the cases when changing the number of leads used.

Hereinafter to the application of the Friedman test, if the null hypothesis is rejected, it is possible to apply another test to obtain a ranking of all the different options. To this end, the Nemenyi test was applied [58]. This test obtained the following ranking (from best to worst): 12, 10, 8, 6, 4 and 2 leads. Other similar tests (Holm [59] and Finner [60]) were also applied and the same ranking was obtained.

## 5. Discussion

In this section, we will discuss and compare the results obtained for heartbeat clustering with PN-EAC on the MIT-BIH Arrhythmia Database and on the INCARTDB.

### 5.1. MIT-BIH Arrhythmia Database

In the results using 25 clusters, despite the slight improvement obtained with the second strategy over the first strategy, from an error of 2.25% to an error of 1.81% (*p* = 0.0821), the differences are not statistically significant. However, the difference between the second and third strategies, which decreases the error from 1.81% to 1.44%, is indeed significant (*p* = 0.0404). This improvement over the second strategy arises from the change in the treatment of the information extracted from the distance between heartbeats, which in the third strategy is used to extract negative evidence. These results support our conjecture that, in some cases, the use of certain information as negative evidence could improve the clustering, hence proving the utility of an algorithm like PN-EAC, which taps both positive and negative evidence.

Using the lifetime criterion, the error between the first and the second strategies increased from 2.75 to 3.81%. At the same time, the total number of clusters is decreased from 508 to 203, which represents a mean of 10.5 and 4.2 clusters per recording, respectively. It is often desirable to get a low number of clusters, since this means that the cardiologists have less work to do, though not at the expense of notably increasing the error. When using the third strategy, the error drops to 0.99%. However, in this case, the number of clusters escalates, creating up to a total of 6947 clusters (a mean of 144.7 clusters per recording). In general, the results of the lifetime criterion were unsatisfactory due to the differences in the number of clusters found in some recordings when using different strategies, and also due to the high number of clusters found in some cases, especially in the third strategy (up to 2977 clusters). The proliferation of clusters, especially in some recordings, seems to be due to the presence of noise and artifacts.

In Table 3, it can be seen that many errors appear in the atrial premature beats (A), which are grouped as normal beats (N) or branch block beats (L and R). The atrial premature beats are distinguished mainly due to the abnormal shape of the P wave. Therefore, it is not surprising that an approach that only takes into account the shape of the QRS complex has problems for recognizing these beats. Another important source of error relies on the nodal escape beats (j); in this case, the beats are again distinguished from the information provided by the P wave and the distances between heartbeats. In PN-EAC, we are only using the information of the distance between heartbeats.

To validate the results obtained with PN-EAC, it is possible to compare them with the results obtained in [12], which is the most referenced work in heartbeat clustering. In [12], authors used self-organizing maps and Hermite functions to cluster the heartbeats, with a fixed number of 25 clusters. The best result obtained in that work is an error of 1.51% for the whole MIT-BIH Arrhythmia Database. This is lower than the results obtained by PN-EAC when using a fixed number of 25 clusters for the first and second strategies (2.15% and 1.81%, respectively). However, the third strategy, which includes the negative evidence, obtains an error of 1.44%, slightly lower than the one obtained in [12]. We could also compare our results with [45], where features extracted from the ECG morphology, heartbeat intervals and RR-intervals, and linear discriminant classifiers were employed to classify five types of heartbeats with an overall error of 14.12%. In [42], a subset of the features presented in [45] and linear discriminant classifiers were used, with which an overall error of 6.11% was obtained.

### 5.2. INCARTDB

**Figure 13:**
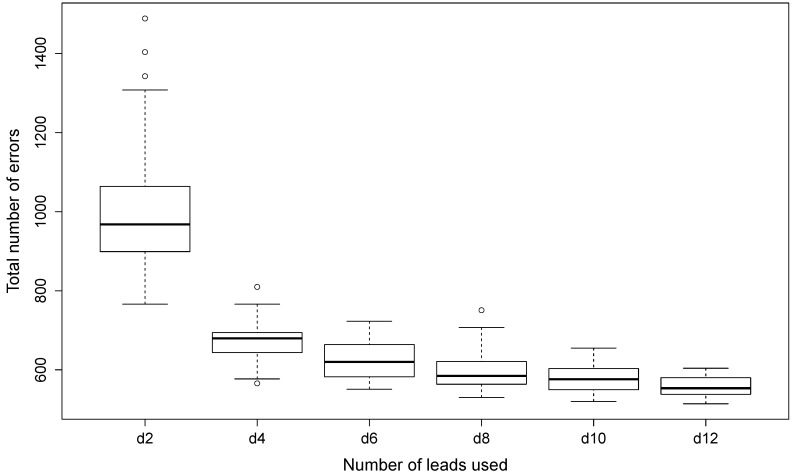
Error results on the St. Petersburg Institute of Cardiological Technics 12-Lead Arrhythmia Database database (INCARTDB) for the different numbers of leads. The outliers are represented as individual points, the boxes are drawn from the value of the first quartile Q1 to the value of the third quartile Q3, corresponding to the horizontal line in the middle to the median (Q2). Vertical lines or “whiskers” are proportional to the difference between Q1 and Q3.

Table 5 shows that the error decreases as the number of leads is increased. The error is reduced from an average of 0.601% using two leads to an average of 0.338% when using the 12 leads. This decrease can be appreciated in Figure 13. The largest decrease is between using two and four leads; further increasing the number of leads keeps dropping the error, but the improvements are smaller.

**Figure 14:**
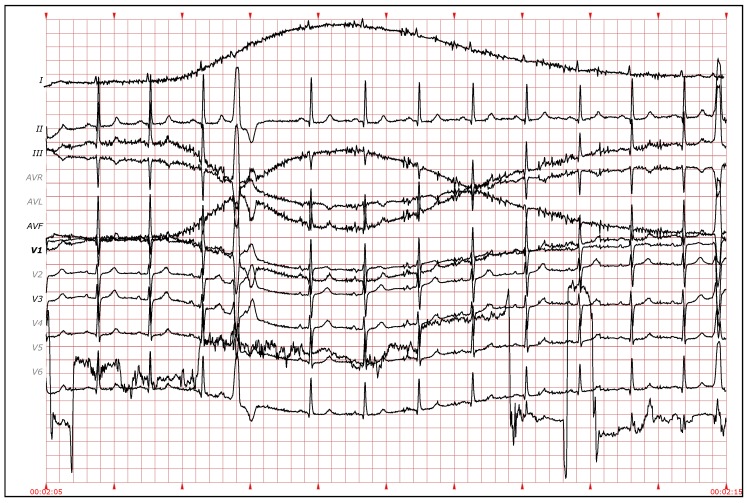
Electrocardiogram (ECG) fragment of 12 leads. Note that some leads, I or V4, have a lot of noise, and it is difficult to distinguish the heartbeats and their shape. (Source: St. Petersburg Institute of Cardiological Technics 12-Lead Arrhythmia Database, recording I66, from 0:02:05 to 0:02:15).

Furthermore, it is possible to see that the variation of the results (interquartile range) is also reduced by adding more leads. The 100 executions also reveal differences according to the specific leads used in that execution (I, II, etc.). This is especially remarkable when using a small number of leads. For example, in the results with two leads, some executions have an error of just 860 heartbeats in all the database, while others reach up to 1400 errors. This variability is reduced when the number of leads increases. This could be due to the bad quality of the signal in some leads (see Figure 14). The evidence obtained from those leads with noise is weak, thus worsening the results of the clustering. When a larger number of leads is used, the relative impact of selecting one of those noisy leads is less and can be compensated by the other leads. Thus, the results of using more leads not only yield higher accuracy, but also more consistence and less variability.

While the INCARTDB has some popularity in the scientific literature, even those authors that use this database, for the most part, only use one [51,61,62,63] or two leads [64]. In [64], two leads are used to develop a linear classifier of heartbeats. This same linear classifier developed in [64] is used in [65], with an extension to 12 leads. Experiments considered the classifier to be applied to all the leads, to a subset of them and to the best lead.

The main reason for not using more than one or two leads is the increment in the dimensionality of the feature space used for the representation of the heartbeat. The concurrent usage of 12 leads implies multiplying by 12 the dimensionality of the feature space, compared to the use of a single lead. This increment of an order of magnitude presents a challenge in the application of machine learning techniques. However, by using the paradigm of evidence accumulation, the PN-EAC algorithm can treat each lead as a complementary independent source of evidence. This solves the problem of the increment of the dimensionality in the feature space, and at the same time permits exploiting and combining all the information of the 12 leads.

In the literature of heartbeat clustering, we have not found any work to compare with the results obtained with PN-EAC in the INCARTDB, nor have we found any work that applies a clustering algorithm over 12 leads of the ECG simultaneously. Based on a clustering technique like the one presented here, it is possible, with the help of a cardiologist who annotates the different heartbeat clusters, to build an assisted classifier [66,67]. The results of this assisted classifier would permit a fair comparison with other classifiers that were applied to the same database.

The work presented in [62] proposes a classifier built from an interactive ensemble learning approach based on the extreme learning machine classifier and the induced ordered weighted averaging operators that obtains, at best, an error of 0.3%. However, to obtain this error, it is necessary for the cardiologist to label at least 200 heartbeats from each recording. In that same work, the best result without labelling any heartbeat (which lends itself to a more direct comparison with the unsupervised approach of the PN-EAC algorithm) presents an error of 23.6%. In [64], features from the RR series, features computed from the ECG samples and different scales of the wavelet transform, along with linear and quadratic discriminant classifiers were employed, and an error of 9.38% was obtained. In [65], using Principal Component Analysis (PCA) over the 12 leads, wavelets to represent the heartbeats and linear discriminant classifiers, an error of 2.88% is obtained. However, [65] divides the heartbeats in only three different classes, while in this work the heartbeats are divided in the five types recommended by the AAMI.

## 6. Conclusions

A new algorithm, named PN-EAC, for data and sensor fusion has been presented in this work. It is a clustering algorithm based on the evidence accumulation paradigm capable of combining and integrating information from multiple sources, while avoiding an increase in the dimensionality of the feature vector. PN-EAC extracts positive evidence from the different data sources to select those elements that should be grouped together in the final partition. Moreover, it combines that positive evidence with negative evidence, which has information about which elements should not be grouped together. The core idea of this concept is that, from certain data sources, it is more reliable to extract information on what elements should not be grouped together (negative evidence) instead of information on what elements should be grouped together (positive evidence). The final partition is obtained from the evidence matrix, which gathers both the positive and negative evidence.

PN-EAC was applied to the problem of heartbeat clustering, to separate the heartbeats according to their type. In the MIT-BIH Arrhythmia Database, three different strategies were applied, obtaining an average error of 1.44% as the best result. This result was obtained by separating the morphological information of the different leads to generate different partitions and deriving negative evidence from the information obtained from the distance between heartbeats. This strategy yielded a statistically significant improvement in performance when compared with the extraction of positive evidence from the distance between heartbeats. These results show the value of the novel concept of negative evidence.

As a general rule, any feature in which dissimilarity could be useful to distinguish between some clusters, but similarity is not useful to group in the same cluster may be considered to extract negative evidence. As we have argued, and shown in the validation over the MIT-BIH Arrhythmia Database, this is the case for the features extracted from the distances between heartbeats for arrhythmia identification. However, we recognize that we cannot provide a precise criterion to determine whether using a feature for the extraction of negative evidence would improve or not the clustering results, beyond the intuitive rule given in this paragraph. Like other steps in the data analysis process, such as determining exactly when a feature has too much noise and it being better to discard it than to include it in the analysis, there is certain subjectivity when making this assessment, and a trial and error process may be necessary as part of the data analysis.

The algorithm was also applied to the St. Petersburg Institute of Cardiological Technics 12-Lead Arrhythmia Database. Using this database, different tests were executed using from 2 to 12 leads. We obtained an average error ranging from 0.601% to 0.338% for 2 and 12 leads, respectively, with a statistically significant decrease in the percentage of error when the number of leads increases. This is due to the fact that each lead provides different nuances of the electrical activity of the myocardium (that is why, in the clinical routine, the 12 leads are usually employed for cardiological diagnosis). This also suggests that, in other ECG databases, like the MIT-BIH Arrhythmia Database, in case the 12 leads were available, PN-EAC would improve its results just by using more leads. These results support the utility of the PN-EAC algorithm as a tool to merge data from multiple data sources. Furthermore, contrary to what usually happens with machine learning techniques, the PN-EAC algorithm does not seem to worsen its performance when there are large amounts of features available, but it improves it. Therefore, this algorithm could be a possible workaround to avoid the problem of the curse of dimensionality in certain problems [68,69]. It is also important to note that the algorithm is highly parallelizable, since the information of the different data sources can be used independently to generate partitions and it is only necessary to combine them at the end to extract the final partition.

Our experience using other algorithms besides K-means to generate the partitions has been mixed. On one hand, when using algorithms with a greater expressive power, like a mixture of models [70], the performance typically increases slightly. However, the execution time increased considerably. On the other hand, using K-means with a larger number of partitions also increases the performance slightly, at the cost of a small increase in the execution time. While we cannot completely rule out that the use of algorithms with more expressive power than K-means could yield better results in some problems, our experience suggests that it is better to generate a greater number of partitions, even though each of these individual partitions is of lower quality, than generating a smaller number of higher quality partitions with a more sophisticated clustering algorithm.

In the future, we would like to apply this algorithm to other fields and different problems that can benefit from combining different sources of information or data fusion. In addition, a dynamic version of the algorithm will be developed to apply it to long or non-ended data streams. This would enable its application to, for example, ECG real-time monitoring. 

## Figures and Tables

**Table 1:** Results of the clustering for the three strategies on the MIT-BIH Arrhythmia Database. ‘1st’ stands for first, ‘2nd’ for second and ‘3rd’ for third. For each strategy and recording (#), the number of errors (Err) using a fixed number of 25 clusters (25C) and using the lifetime criterion are shown. In the latter, the selected number of clusters is also shown. At the end of the table, the total number of errors (***Total Err.***) and the percentage of error (***% Err.***) over all the heartbeats in the database are shown.

#	1st Strategy			2nd Strategy			3rd Strategy		
	25C	Lifetime		25C	Lifetime		25C	Lifetime	
	Err	Err	Clusters	Err	Err	Clusters	Err	Err	Clusters
100	33	33	5	6	33	2	9	33	5
101	3	3	7	0	3	5	1	3	5
102	7	47	6	13	58	4	28	28	20
103	1	1	15	0	1	2	0	0	81
104	251	257	11	309	351	4	110	106	43
105	11	12	10	5	5	5	5	5	52
106	2	10	9	1	28	7	0	0	85
107	0	1	9	1	1	3	0	0	8
108	11	16	9	9	9	2	6	6	66
109	4	9	14	2	10	2	2	2	39
111	0	0	11	0	0	2	0	0	22
112	2	2	7	1	2	3	1	1	16
113	0	0	11	0	0	2	0	0	49
114	12	16	12	11	16	4	13	6	61
115	0	0	4	0	0	3	0	0	5
116	2	2	13	0	2	2	1	1	11
117	1	1	7	0	0	7	1	1	15
118	96	96	12	58	100	2	34	12	141
119	0	0	7	0	0	2	0	0	33
121	1	1	12	0	1	2	1	1	5
122	0	0	8	0	0	8	0	0	4
123	0	0	6	0	0	2	0	0	16
124	36	43	9	41	41	4	38	36	44
200	129	130	15	117	531	14	84	84	22
201	48	54	7	50	65	4	44	44	21
202	37	42	12	17	56	2	23	23	23
203	81	82	19	286	385	2	75	0	2977
205	14	14	9	13	15	6	13	15	16
207	187	196	20	52	318	5	133	17	64
208	107	109	17	120	449	3	96	98	21
209	181	298	5	106	162	3	66	65	31
210	32	37	17	30	71	2	33	26	57
212	0	0	11	3	4	4	1	0	43
213	112	351	8	90	396	3	126	126	29
214	6	6	14	4	5	12	3	3	73
215	4	5	20	9	26	3	4	4	17
217	34	50	10	65	69	10	4	3	47
219	11	12	9	11	18	3	11	11	18
220	94	94	3	4	94	2	30	94	2
221	1	3	8	1	1	6	0	0	41
222	389	389	10	328	421	2	338	0	2481
223	116	125	17	108	265	3	106	77	32
228	3	3	14	3	4	7	3	3	10
230	0	0	9	0	0	3	0	0	12
231	2	2	5	2	2	5	2	2	5
232	388	398	12	80	89	15	123	136	11
233	19	20	18	32	35	3	16	14	60
234	2	50	5	1	50	2	1	1	8
***Total Err.***	2470	3020	508	1989	4192	203	1585	1087	6947
***% Err.***	2.25	2.75		1.81	3.81		1.44	0.99	

**Table 2:** *P*-values of the Wilcoxon test for the different strategies on the MIT-BIH Arrhythmia Database. ‘1st’ stands for first, ‘2nd’ for second, ‘3rd’ for third, ‘vs.’ for versus and ‘Stra.’ for strategy.

	1st Stra. vs. 2nd Stra.	1st Stra. vs. 3rd Stra.	2nd Stra. vs. 3rd Stra.
**25 Clusters**	0.0821	<0.0001	0.0404
**Lifetime Criterion**	0.0046	<0.0001	<0.0001

**Table 3:** Confusion matrix for the third strategy on the MIT-BIH Arrhythmia Database using the original annotations of the database. ‘Se’ stands for sensitivity and ‘P+’ for positive predictivity value.

	N	L	R	a	V	F	J	A	S	E	j	P	Q	!	e	f
**N**	74,920	0	1	9	125	106	4	395	2	1	177	0	11	0	16	11
**L**	0	8072	1	0	2	2	0	106	0	0	0	0	2	1	0	0
**R**	1	0	7212	0	0	3	29	118	0	2	5	0	0	0	0	0
**a**	2	0	0	132	1	1	0	3	0	0	0	0	0	0	0	0
**V**	26	0	0	4	6906	39	0	20	0	0	0	0	1	13	0	0
**F**	13	0	0	0	91	652	0	0	0	0	0	0	1	0	0	0
**J**	1	0	0	0	0	0	50	0	0	0	0	0	0	0	0	0
**A**	37	0	41	5	0	0	0	1897	0	0	2	0	0	0	0	0
**S**	0	0	0	0	0	0	0	0	0	0	0	0	0	0	0	0
**E**	0	0	1	0	0	0	0	0	0	101	0	0	0	1	0	0
**j**	14	0	0	0	0	0	0	5	0	0	45	0	0	0	0	0
**P**	0	0	0	0	1	0	0	0	0	0	0	6956	1	0	0	43
**Q**	0	0	0	0	0	0	0	0	0	0	0	0	5	0	0	0
**!**	0	0	0	0	4	0	0	0	0	2	0	0	0	457	0	0
**e**	0	0	0	0	0	0	0	0	0	0	0	0	0	0	0	0
**f**	2	0	0	0	0	0	0	0	0	0	0	68	12	0	0	928
**Se**	99.87	100.00	99.39	88.00	96.86	81.20	60.24	74.57	0.00	95.28	19.65	99.03	15.15	96.82	0.00	94.50
**P+**	98.87	98.61	97.86	94.96	98.53	86.13	98.04	95.71	-	98.06	70.31	99.36	100.00	98.70	-	91.88

**Table 4:** Confusion matrix for the third strategy on the MIT-BIH Arrhythmia Database using the annotations recommended by the Association for the Advancement of Medical Instrumentation (AAMI). ‘Se’ stands for sensitivity and ‘P+’ for positive predictivity value.

	N	S	V	F	Q
**N**	90,201	855	129	111	24
**S**	100	2147	3	1	0
**V**	28	22	7484	39	1
**F**	13	0	91	652	1
**Q**	2	0	1	0	8013
**Se(%)**	99.84	71.00	97.09	81.20	99.68
**P+(%)**	98.77	95.38	98.81	86.13	99.96

**Table 5:** Clustering results on the St. Petersburg Institute of Cardiological Technics 12-Lead Arrhythmia Database database (INCARTDB) for 2 (d2), 4 (d4), 6 (d6), 8 (d8), 10 (d10) and 12 (d12) leads. For each recording, the average number of errors of the 100 executions is shown. At the end of the table, the mean of the average number of errors (***Avg. Err.***) and the percentage of error (***% Err.***) for the 100 executions over all the heartbeats in the database are shown.

	d2	d4	d6	d8	d10	d12
I01	0.75	0.07	0	0	0	0
I04	33.79	31.87	30.42	28.58	28.42	27.89
I05	15.73	13.66	13.24	12.73	11.38	10.87
I06	23.35	13.84	10.05	9.44	9.47	9
I07	19.17	5.22	3.72	3.6	3.33	3.07
I08	6.35	3.64	3.76	3.13	3	2.37
I09	9.76	9.51	9.16	9.15	8.67	8
I10	0.85	0.2	0.01	0.01	0	0
I11	24.02	24	24	24	24	24
I12	3.97	2.99	3.11	2.96	3.05	3.15
I13	0	0	0	0	0	0
I14	0	0	0	0	0	0
I15	0.04	0	0	0	0	0
I16	0.04	0	0	0	0	0
I17	0.19	0.01	0	0	0	0
I18	57.08	52.74	51.52	50.54	50.37	51.7
I19	1.77	1.5	1.43	1.35	1.51	1.82
I20	102.1	60.59	52.78	47.6	47	39.87
I21	51.95	26.31	25.4	24.29	22.59	22.28
I22	63.79	41.17	37.23	36.81	35.93	37.31
I23	0.03	0	0	0	0	0
I24	0.08	0	0	0	0	0
I25	1.57	1.05	1.14	1	1	1
I26	3.8	2.22	2.22	1.96	1.98	2
I27	0.01	0	0	0	0	0
I28	0	0	0	0	0	0
I29	10.55	2.09	1.23	0.61	0.35	0.12
I30	1.9	0.41	0.05	0	0	0
I31	41.17	35.25	45.28	33.29	29.44	19.85
I32	0.06	0	0	0	0	0
I33	90.59	26.28	16.7	15.36	15.07	15.3
I34	63.73	15.93	11.89	11.51	11.85	11.93
I35	59.05	45.68	41.64	37.67	34.94	34.96
I36	47.48	38.37	34.69	36.3	36.67	42.15
I37	1.02	1	1	1	1	1
I38	2.37	0.87	0.64	0.73	0.57	0.33
I39	1.68	0.15	0.03	0.01	0	0
I40	4.26	3.42	3.46	3.23	3.18	3.17
I41	4.96	4.95	4.94	4.82	4.93	5
I42	5.98	5.64	5.19	5.33	5.51	5.18
I43	7.05	5.96	5.07	5.01	5	5
I44	7.04	5.75	5.68	5.48	4.82	4.17
I45	0.02	0	0	0	0	0
I46	7.88	6.02	5.75	5.39	4.97	4.97
I47	2.86	1.12	1.07	1.03	1.02	1
I48	3.15	2.53	2.43	2.66	2.78	3
I49	0	0	0	0	0	0
I50	1.91	1.85	1.72	1.82	1.83	1.12
I51	3.34	3.09	2.88	3.01	2.93	3
I52	0	0	0	0	0	0
I53	0.1	0.03	0	0	0	0
I54	2.85	0.31	0.16	0.11	0.02	0
I55	1.47	0.45	0.09	0.04	0	0
I56	1.71	0.06	0.02	0	0	0
I59	38.17	37.62	37.83	34.87	33.96	33.05
I60	50.23	48.86	48.58	47.21	45.28	42.41
I61	0.88	0.89	0.89	0.93	0.98	1
I62	36.72	26.77	25.6	24.22	23.49	21.92
I63	12.42	10.68	9.72	8.74	9.4	9.48
I64	2.43	1.94	2.2	1.93	1.96	2
I65	11.6	9.47	8.84	8.84	8.94	8.98
I66	1.81	1.77	1.91	1.85	1.81	2
I67	6.15	5.05	5	5	5	5
I68	1.37	1.01	1	1	1	1
I69	0.71	0.37	0.66	0.58	0.76	0.83
I70	0.01	0	0	0	0	0
I71	9.08	6.49	5.97	5.85	5.72	5.93
I72	7.77	7.46	7.56	7.54	7.86	8
I73	3.93	2.02	2.51	2.02	2	2
I74	15.11	12.37	11.66	10.35	11.46	10.04
I75	1.42	0.17	0.03	0	0	0
***Avg. Err.***	994.2	670.7	630.8	592.5	578.2	559.2
***% Err.***	0.601	0.405	0.381	0.358	0.349	0.338

## Abbreviations

The following abbreviations are used in this manuscript:

PN-EAC	Positive and Negative Evidence Accumulation Clustering
MIT-BIH	MIT-BIH Arrhythmia Database
INCARTDB	St. Petersburg Institute of Cardiological Technics 12-Lead Arrhythmia Database
ECG	Electrocardiogram
EAC	Evidence Accumulation Clustering
AAMI	Association for the Advancement of Medical Instrumentation
PCA	Principal Component Analysis
